# The Efficacy of Clove Oil Against *Aspergillus flavus* and the Production of Aflatoxin B1 in Organic Peanuts in Georgia

**DOI:** 10.1021/acsomega.5c06467

**Published:** 2025-10-06

**Authors:** Ari J. Schwartz, Md Ackas Ali, Mohammad A. Halim, Premila N. Achar

**Affiliations:** † Department of Molecular and Cellular Biology, 15617 Kennesaw State University, Kennesaw, Georgia 30144, United States; ‡ Department of Chemistry and Biochemistry, Kennesaw State University, Kennesaw, Georgia 30144, United States

## Abstract

Georgia produces half of the United States’ peanuts, but the Southeast loses 25 million U.S. dollars annually due to aflatoxins from *Aspergillus* spp. contamination. *Aspergillus flavus* produces aflatoxin B1 (AFB1), one of the most toxic and carcinogenic substances affecting humans, livestock, and crops globally. Synthetic chemicals, genetic engineering, and biological control methods have been employed against *A. flavus* with limited success. Recently, there has been considerable interest in using plant-based antimicrobial compounds against mold. Our previous study established the aflatoxigenic properties of clove essential oil (EO) against *A. flavus*. We now evaluate the effectiveness of clove EO in artificially inoculated organic peanuts. Tween 20 served as a control, and all experiments were done in replicates. AFB1 was quantified using selected ion monitoring (SIM) based liquid chromatography–mass spectrometry (LC–MS) techniques. Our findings demonstrated that a high concentration of clove EO significantly reduced AFB1 in infected peanuts. Interestingly, a high reduction in AFB1 production was observed between 0 and 500 ppm, and the reduction increased slightly at higher concentrations of 1000 and 2000 ppm, respectively. Our results demonstrate that clove EO is most effective at reducing AFB1 in infected peanuts at low concentrations. We will further investigate the potential of clove EO as a biological control agent against *A. flavus*.

## Graphical abstract

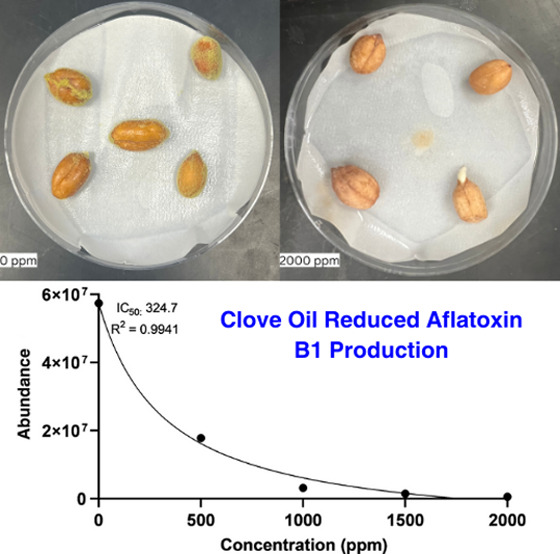

## 1 Introduction

Peanuts are the world’s fourth most important source of edible vegetable oil and the third most important source of vegetable protein feed meal, and Georgia produces more than 50% of the United States’ peanuts. Approximately 25 million U.S. dollars of peanuts are lost in agriculture annually due to contamination by *Aspergillus* spp., making it a costly problem for the agricultural industry.^1–3^ In 2019, more than 117 million U.S. dollars were lost, amounting to 84 U.S. dollars per acre.^4^ Lawley^5^ reported that *Aspergillus flavus* is the most common species that contaminates peanuts by producing carcinogenic aflatoxins, which destroy peanut shells before they are harvested. These fungi also produce aflatoxins, which are secondary metabolites that are both highly toxic and carcinogenic, posing a significant threat to humans and livestock.^6^ AFB1 remains the most common and reportedly the most toxic substances^7,8^ and has been classified as a Class 1 human carcinogen by the International Agency for Research on Cancer (IARC) since it induces the formation of DNA adducts that contribute to liver cancer formation.^9,10^ Despite the recognized impact and consequences of aflatoxin-producing fungi in peanut production, measures to address this global issue remain limited.^11,12^ Current strategies to control *A. flavus* heavily depend on synthetic fungicides and preservatives, such as benzimidazoles, which may produce side effects, including carcinogenicity, teratogenicity, and toxicity to consumers when used excessively.^13^ Second, these fungicides require advanced specialized equipment and expensive chemicals or reagents.^14–16^ Third, the use of benzimidazoles and their derivatives contributes to environmental pollution.^17^ The extensive use of these substances may produce several side effects in consumers and increase the risk of high-level toxic residues in food products.^18^ Several field control measures are being utilized or explored, including modifying cultural practices, developing resistant cultivars, competitive exclusion using strains that do not produce aflatoxin, and the development of field treatments that block aflatoxin production.^19^ Thus, alternative plant antimicrobial agents to control toxigenic *Aspergillus* spp. are gaining global interest among researchers.^20,21^

Natural products, such as EOs, could potentially serve as effective alternatives to synthetic chemicals for controlling food contamination by *Aspergillus* spp.^22^ Abd El-Aziz et al.^23^ identified cinnamon and thyme EOs as having more substantial antimicrobial potential in cashews than garlic, mint, and rosemary. Similarly, Xiang et al.^24^ found that 11 EOs such as cinnamon, oregano, lemongrass, clove, citronella, basil, thyme, *Litsea cubeba*, peppermint, mugwort, and rosemary showed antiaflatoxigenic activities against in maize. Juglal et al.^25^ evaluated the effects of nine different oils on the growth of *Aspergillus parasiticus* and *Fusarium moniliforme*, identifying clove EO as the most inhibitory, followed by cinnamon, oregano, and mace oils, while neem and eucalyptus EOs showed no effect on fungal growth. They also observed that incorporating cloves into mycotoxin-infected grain significantly reduced aflatoxin contamination, suggesting that clove EO could effectively control mycotoxigenic fungi and prevent toxin formation, offering a simple measure for rural communities to prevent toxin contamination in grains. Atwa et al.^26^ investigated the antifungal properties of mint and clove EOs on peanuts, aflatoxin production, and yield. They reported that treatment with mint and clove EOs significantly reduced aflatoxin levels compared to chemicals, while decreasing total fungal counts in both seasons. They determined significant interactions between different peanut genotypes and treatments in the Gregory peanut strain, with mint oil showing the highest values across various agronomic traits.^26^ Achar et al.^6^ demonstrated for the first time the implication of plant-based antimicrobial compounds against *A. flavus* and the production of AFB1 in Georgia peanuts, variety Tifguard. The demand to reduce the contamination of peanuts by *A. flavus* in Georgia and all peanut-growing states in the United States continues to grow. Additionally, the primary reason for considering EOs as antifungal agents is that they are classified as generally recognized as safe (GRAS). Moreover, there is a global interest in using plant-based antimicrobial compounds that are GRAS.^6^

Several methods have been used to quantify AFB1 in seeds. Traditional methods for detecting AFB1 content, such as high-performance liquid chromatography (HPLC), thin-layer chromatography (TLC), and enzyme-linked immunosorbent assay (ELISA), offer the advantages of a low limit of detection (LOD) and reasonable specificity. However, these methods are time-consuming, labor-intensive, complicated, and destructive to samples. As a result, these methods are unsuitable for detecting AFB1 in peanuts in batches at postharvest, either in storage or food processing facilities.^27^ These authors also utilized short-wave infrared (SWIR) hyperspectral imaging (HIS) to detect AFB1 contamination in peanut seeds, indicating that this technique, in combination with scanning electron microscopy (SEM) and transmission electron microscopy (TEM), has excellent potential for AFB1 quantification.^26^

In our previous study, we screened 15 EOs and investigated their antifungal properties against *A. flavus* from contaminated peanuts using HPLC to quantify AFB1. We also concluded that clove EO could be a promising natural fungicide for effective biocontrol, a nontoxic biopreservative, and an eco-friendly alternative to synthetic additives against *A. flavus* in Georgia peanuts.^6^ In this study, however, we focus on the efficacy of clove EO in contaminated organic peanut seeds in reducing AFB1. LC–MS in combination with SIM is implemented for quantitative metabolomics profiling of AFB1 in organic peanuts. We further investigated whether this sustainable, and eco-friendly biological control agent, such as clove EO, can also control *A. flavus* in the organic peanuts and reduce AFB1 production. Our results may certainly benefit the organic farming community in the state of Georgia.

## 2 Materials and Methods

### 2.1 Isolation of *A. flavus* from Peanuts

From our previous study,^6^ peanut seeds, variety Tifguard (runner-type), courtesy of Agricultural Research Service (ARS), Tifton, Georgia, US, were incubated on moist filter paper for 7 days. *A. flavus* was isolated from contaminated peanuts and was plated onto a potato dextrose agar (PDA) medium (Fisher Scientific, Waltham, MA, USA). The plates were incubated with alternating periods of 12 h of light and darkness for 7 days. In addition, isolates from the contaminated peanuts were compared to standard strains of *A. flavus* (ATCC 11498), American Type Culture Collection. The fungal colonies were observed under a light microscope (Leica, M13595, Leica Microsystems, Wetzlar, Germany) and identified based on their morphological characteristics, such as the color of the colony, conidial heads, vesicles, phialides, and conidia, using fungal keys and manuals.^5,19,28–31^ A standard spore suspension of *A. flavus* was freshly prepared by transferring a loopful of spores from a five-day-old pure culture plate into 5 mL of sterile water. The cell concentration of 10^6^/mL in the photo calorimeter was adjusted by further dilution with sterile water, ensuring the suspension’s optical density (OD) was 0.01 at 460 nm. Yeast extract sucrose (YES) agar was used as a medium to enhance AFB1 production.^32^ The isolated *A. flavus* was inoculated onto this medium, and the plates were sealed and incubated at 27 °C in a CO_2_ incubator (Fisher Scientific, Isotemp, Waltham, MA, USA) for 10–15 days. Following incubation, the plates were observed under ultraviolet light (UV) (Spectroline CC-80, Fisher Scientific, Waltham, MA, USA) to detect the presence of AFB1 production. If the mold fluoresced under UV light, it was considered aflatoxin-positive and confirmed as an aflatoxigenic form of *A. flavus*.

### 2.2 Subculturing *A. flavus* in Rose Bengal Media

Rose Bengal Agar (31.55 mg) was prepared by the manufacturer’s (Fisher Scientific) instructions in 1000 mL of DI water. Petri plates were inoculated with *A. flavus* (Phase 2.1), sealed with parafilm for 5 days, and stored in the refrigerator at 4 °C.

### 2.3 Selection of Plant-Based EOs

Following our previous study,^6^ cloves, which showed maximum antifungal and antiaflatoxigenic activities, were selected for the current investigation. Clove (*Syzygium aromaticum*), 100% pure according to the manufacturer (Sigma-Aldrich, USA), was used. A stock concentration of clove EO (50,000 ppm) was emulsified with 0.5% Tween 20 (v/v) for further use.

### 2.4 Artificial Inoculation of Peanut Seeds

Organic peanut variety, GA Greener, was selected as the subject of our study. A group of 100 sizable, deshelled seeds without skin damage was surface sterilized with 1% sodium hypochlorite and vortexed for 1 min. Samples were soaked and rinsed with sterile water three times. The surface moisture of the kernels was air-dried and wiped with Kimwipes. A spore suspension of *A. flavus* was prepared (10^6^ spores in 1 mL of water) from cultures grown on Rose Bengal medium and mixed thoroughly. The seeds were artificially inoculated with 1 mL of spore suspension. Seed samples were incubated at 21 °C for 8 days in a laminar flow hood in a beaker sealed with parafilm, and the latter was punctured for aeration. Sterile water was added to the beaker and shaken on day 4 of incubation to ensure the peanuts remain hydrated.

### 2.5 Exposure of Peanut Seeds with Clove EO

Only 75 of the 100 artificially inoculated seeds (Phase 2.4) were emersed in flasks containing varying concentrations of clove EO (500, 1000, 1500, and 2000 ppm) from the stock solution containing Tween 20 (Phase 2.3) and sealed with parafilm. Subsequently, treated and untreated samples were placed in a shaker incubator at 180 rpm for 24 h.

### 2.6 Plating Clove EO Treated Peanut Seeds

Following incubation, all seeds were plated equidistantly (5 seeds/plate) on moist filter paper in Petri plates and incubated for 8 days with alternating 12 h periods of light and darkness at 21 °C in a laminar flow hood. Sterilized water was used to ensure adequate moisture during the incubation process. Seeds contaminated with molds other than *A. flavus* were placed into their own plates.

### 2.7 Preparation of Samples for Quantification of AFB1

The seeds from each treatment group were ground into 0.2 g pastes using a mortar and pestle. The seed pastes were rapidly frozen by quenching them in liquid nitrogen for 5 min. Each set of frozen paste was homogenized with 14 mL of methanol and centrifuged at 7800 rpm for 10 min to obtain the precipitate. The supernatant was vacuum centrifuged to evaporate the methanol under reduced pressure, thereby extracting the metabolites. Samples were resuspended in 100 μL of 0.1% formic acid in water for LC–MS analysis to quantify all samples. The data collected were plotted as a mass spectrum for each control group of peanuts treated with varying concentrations of clove EO as indicated (Phase 2.5) and as shown (Figure 1a–e) using Origin 2024b software.

**Figure 1:**
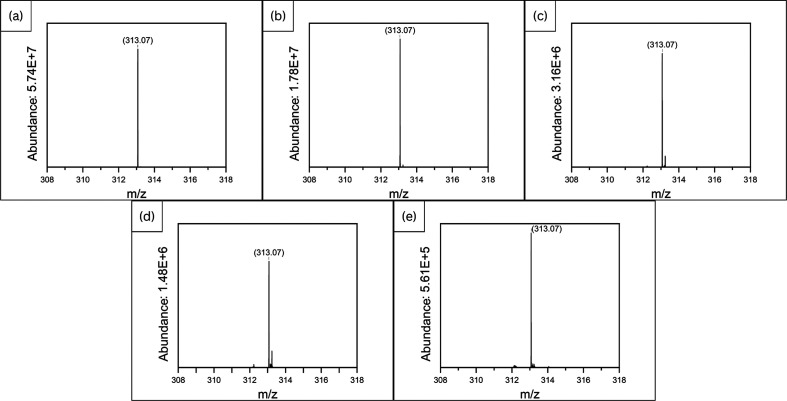
Mass spectrum of AFB1 produced by organic peanuts treated with varying concentrations of clove EO (a) 0 ppm; (b) 500 ppm; (c) 1000 ppm; (d) 1500 ppm; and (e) 2000 ppm.

### 2.8 AFB1 Quantification in Mycelium and Spores Using SIM-Based LC–MS

The chromatographic analysis in this study was conducted using a Vanquish HPLC system. The separation procedure utilized an Accucore RP-MS HPLC column. A and B were water and acetonitrile, respectively, with 0.1% formic acid (FA) added to the mobile phase A 12 min gradient elution program was developed, beginning with 2% B for 1 min; increasing linearly to 40% B from 1 to 6 min; 40% to 60% B from 6 to 8 min; then 60% to 90% B from 8 to 9 min; holding at 90% B from 9 to 10 min, and finally decreasing to 2% B from 10 to 12 min. The Orbitrap Exploris 240 mass spectrometer was used to determine the samples in positive ionization mode.

### 2.9 Calibration Curve of AFB1 Standard

A reference curve was created using standard AFB1 at concentrations of 0.5, 1, 5, 10, and 20 ppm (Figure 2a). The calibration curve was plotted using GraphPad Prism software. In addition, data collected from 20 ppm was plotted as a chromatogram (Figure 2b) and a mass spectrum (Figure 2c).

**Figure 2:**
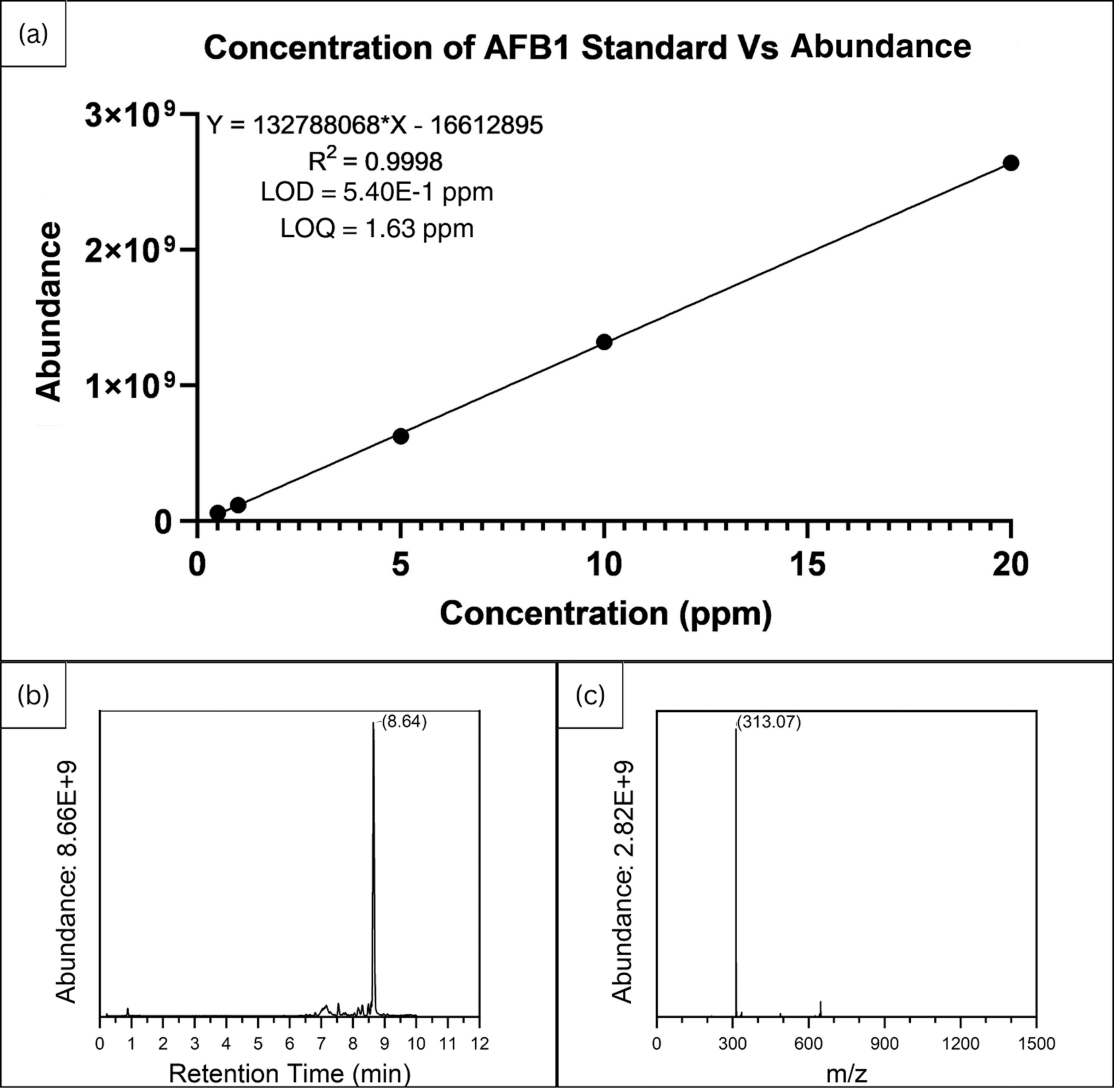
(a) Calibration curve of AFB1 standard. (b) Chromatogram of AFB1 Standard (20 ppm). (c) Mass spectrum of AFB1 Standard (20 ppm).

### 2.10 Statistical Analysis

The data were statistically analyzed and presented as an inhibition curve and a column plot using GraphPad Prism software. The IC_50_ value, representing the concentration (ppm) of clove EO required to inhibit 50% of AFB1 abundance in treated peanuts, was calculated, and the corresponding *R* ^2^ value was obtained.

## 3 Results

### 3.1 Subculturing *A. flavus* in Rose Bengal Media

We observed *A. flavus* isolated from contaminated peanuts in Rose Bengal Agar. We could clearly see progressive growth from day 1 of incubation to day 5, showing mycelium with abundant asexual spores (Figure 3).

**Figure 3:**
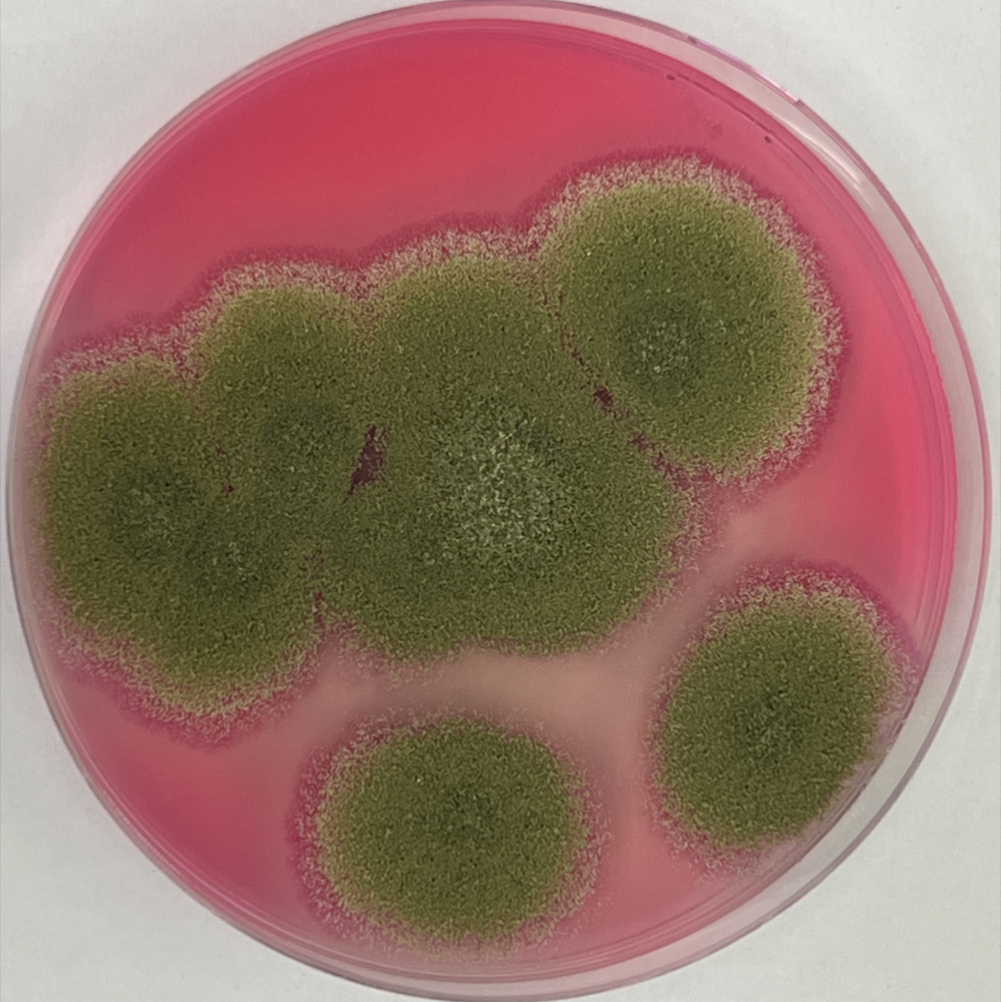
Growth of mycelium and spores of *A. flavus* on Rose Bengal Agar.

### 3.2 *A. flavus* Inoculated Peanut Seeds and Clove EO Treatment

After 8 days of incubation, the clove EO-treated seeds showed varying degrees of infection by *A. flavus*. There was a progressive decrease in *A. flavus* contamination with increasing concentration of clove EO (Figure 4).

**Figure 4:**
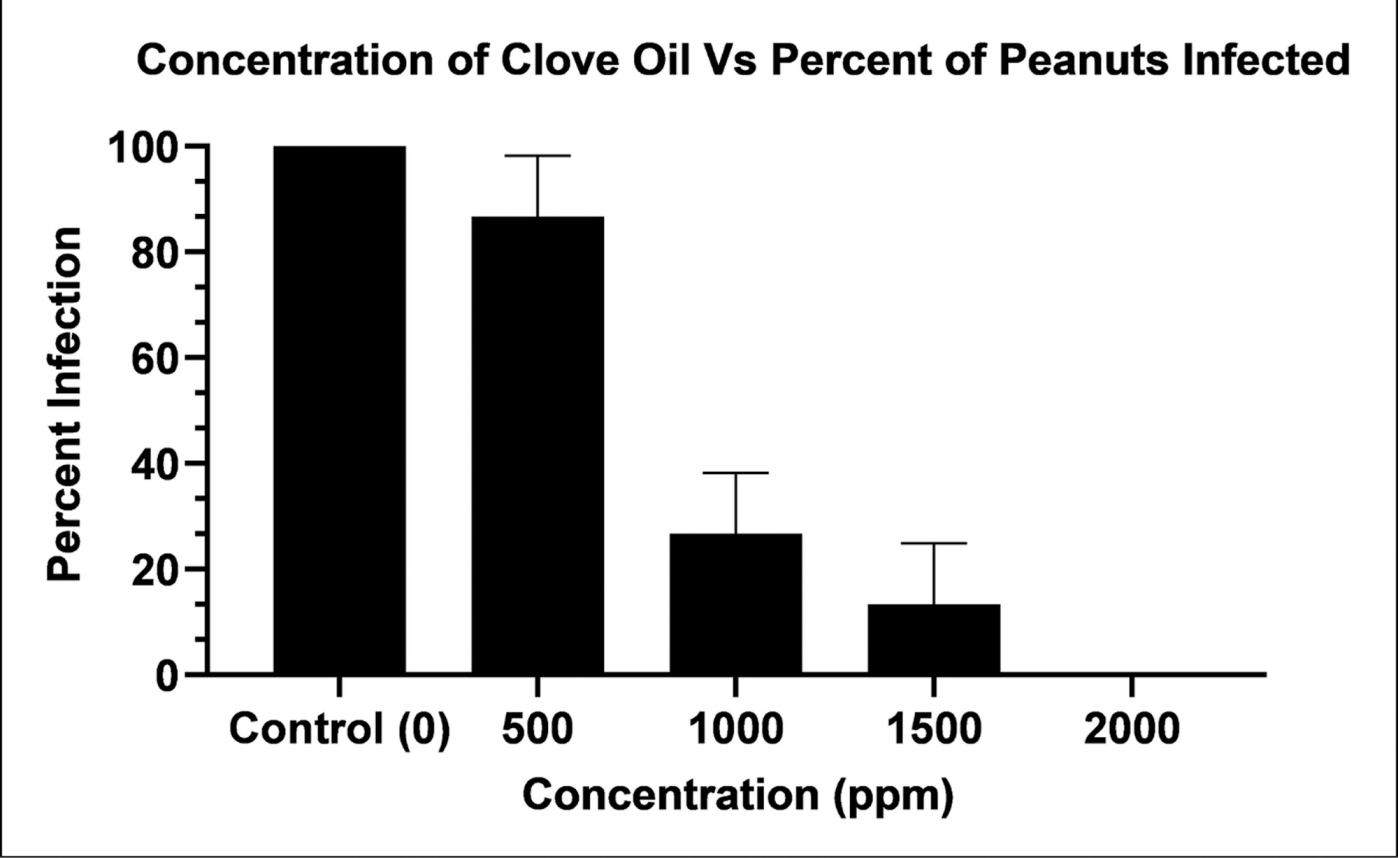
One-way ANOVA column plot of clove EO (ppm) in treated organic peanuts versus the (%) infection in organic peanuts with apparent *A. flavus* infection.

The seeds treated with the highest concentration (2000 ppm) had no visible growth of *A. flavus* (Figure 5e), while all EO-untreated seeds showed contamination (Figure 5a).

**Figure 5:**
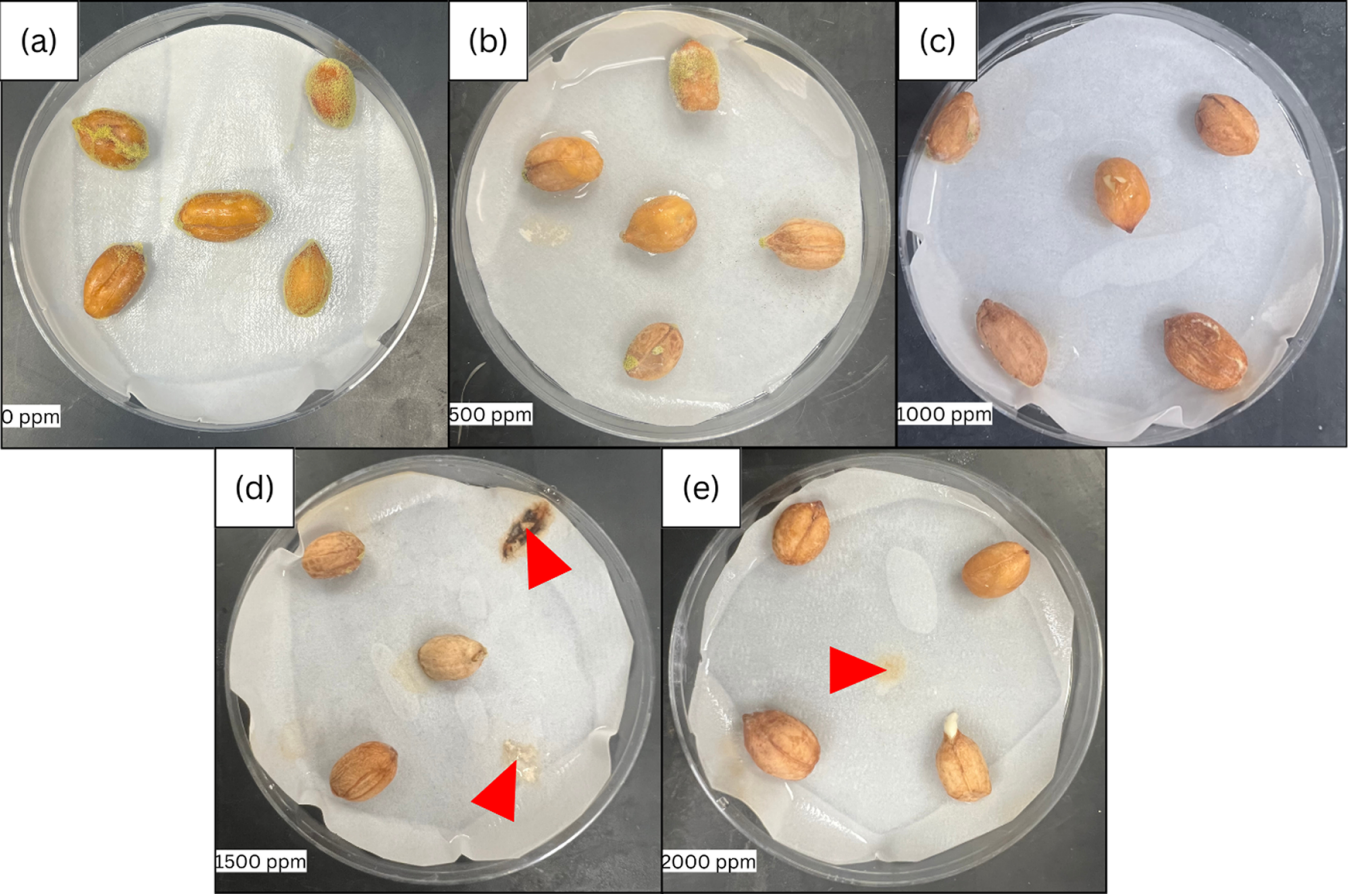
Decrease of *A. flavus* growth in organic peanut seeds with increase in concentrations of clove EO (a) 0 ppm; (b) 500 ppm; (c) 1000 ppm; (d) 1500 ppm; and (e) 2000 ppm. Arrows indicate removed seeds contaminated with molds other than *A. flavus*.

### 3.3 Quantification of AFB1 from Clove EO-Treated Peanut Seeds

Our LC–MS analysis on the effect of various concentrations of clove EO on the production of AFB1 by *A. flavus* in contaminated seeds (Figure 1a–e) and a standard of AFB1 (Figure 2c) showed the highest peak intensity (abundance) of AFB1 at *m*/*z* 313.07. The *m*/*z* values obtained from the mass spectrum match with a singly protonated molecular weight of AFB1 (313.27 g/mol).^33^ After plotting the data of the inhibition curves using GraphPad Prism software, we also observed a dose-dependent inhibition of AFB1 production by clove EO. The abundance of AFB1 decreased significantly with increasing concentrations of clove EO (Figure 6). The seed samples treated with 2000 ppm of clove EO demonstrated the lowest abundance of 5.62 × 10^5^, indicating a significant decrease in AFB1 levels. In contrast, the control sample (0 ppm) displayed the highest abundance of 5.74 × 10^7^. The inhibitory effect of clove EOs on AFB1 production was further analyzed by determining the half-maximal inhibitory concentration values (IC_50_). The IC_50_ value for the clove EO was determined to be 324.7 ppm, with a decent coefficient of determination (*R* ^2^ = 0.9941) (Figure 6). Additionally, we observed that AFB1 production was significantly reduced between 0 and 500 ppm of clove EO.

**Figure 6:**
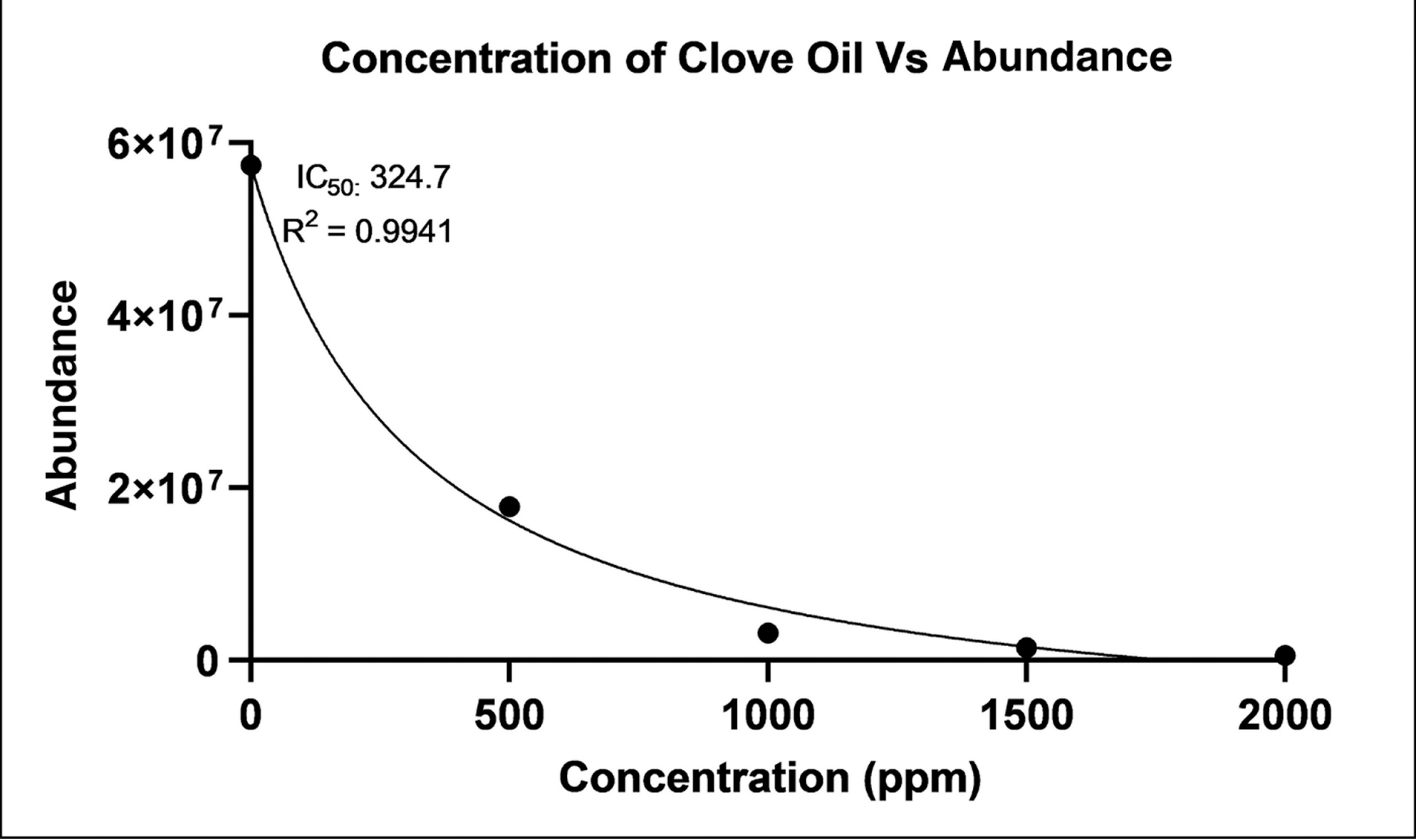
Inhibition curve of clove EO (ppm) in treated organic peanuts versus AFB1 abundance.

## 4 Discussion

Peanut infection by *A. flavus* and aflatoxin problems persist in Georgia, resulting in significant economic losses. It has been recorded globally that controlling aflatoxin is crucial due to its detrimental effects on human and animal health worldwide.^34^ Several strategies have been developed to minimize aflatoxin contamination in crops, including traditional and innovative techniques to control *Aspergillus* spp. in food and feed,^7^ and chemicals have been reported to adversely affect the environment by causing toxic build-up, infertility, and groundwater contamination.^35^ However, reports on the antifungal properties of EOs, with evidence of their historical and long-term use in human health and food settings for preventing and managing fungal infections, are emerging.^36–41^ Based on our previous report^6^ on the antifungal activities of EOs against *A. flavus* in Georgia peanuts, we have now tested the efficacy of clove EO as an antifungal and antiaflatoxigenic agent against *A. flavus* in organic peanut seeds.

In our current study, seeds treated with the highest concentration of clove EO (2000 ppm) showed no visible mycelial growth (Figure 5e). In contrast, all untreated seeds exhibited growth of mycelium and spores (Figure 5a). Our results corroborate with the observations reported by Gemeda et al.,^42^ who found that Ajwain EO exhibited absolute mycelial inhibition of *Aspergillus niger* grown on Sabouraud dextrose broth at a concentration of 1000 ppm and absolute spore inhibition at 2000 ppm. This consistency is further evident in similar studies, such as those of Abd El-Aziz et al.,^23^ who observed that similar to cinnamon EO (4%) and thyme (4%) inhibited the growth of *A. flavus* dry weight mycelium from cashews, supporting the notion that EOs can serve as antifungal agents against *Aspergillus* spp., and potentially reducing the reliance on synthetic fungicides. Similarly, in our previous antifungal inhibition assay screening 15 EOs at concentrations ranging from 0 to 2000 ppm against *A. flavus*,^6^ clove EO was found to be the most effective, achieving total mycelial inhibition at 500 ppm and over 90% inhibition at 250 ppm, highlighting its concentration-dependent efficacy. These findings align closely with the in vitro and in vivo results reported for *Cinnamomum burmannii* leaf EO (YXYO), which significantly inhibited *A. flavus* mycelial growth and completely suppressed AFB1 production at a concentration of 30 μL/disc. The study demonstrated that colony diameter inhibition increased from 36.96% to 80.94% with rising YXYO concentrations, and mycelial dry weight decreased correspondingly.^43^ However, our study focused exclusively on organic peanuts in order to support the sustainability and economic viability of organic peanut farmers in Georgia.

The antiaflatoxigenic characteristics of clove EO against *A. flavus* in organic peanuts were also investigated in this study. Using selected ion monitoring (SIM)-based liquid chromatography–mass spectrometry (LC–MS), we measured AFB1 concentrations in the mycelium and spores. We used an AFB1 standard as a reference for comparison. Our findings demonstrated the growth inhibition of *A. flavus* when treated with clove EO. We also demonstrated an explicit dose-dependent inhibition of AFB1 production by clove EO, however, clove EO inhibited with diminishing efficacy (Figure 6). The decrease in AFB1 corresponds to the inhibition of *A. flavus* growth, but there is still the possibility of metabolic inhibition of AFB1. Both our findings support the previous report of Mabrouk and El-Shayeb^44^ who quantified mycelial growth by dry weight measurements and quantified aflatoxin production using TLC on silica gel G and spectrophotometry, and found that clove EO inhibited mycelial growth of *A. flavus* and aflatoxin production in seeds such as rice powder-corn steep (RC) medium at concentrations above 1000 ppm. Thus, supporting the idea that EOs not only inhibit mycelial growth but also inhibit the production of aflatoxins. Similarly, Abd El-Aziz et al.^23^ tested other EOs, such as cinnamon, garlic, mint, rosemary, and thyme, to control aflatoxins in cashew nuts contaminated by *A. flavus* and *A. parasiticus*. Their HPLC analysis showed that AFB1 and AFB2 produced by both these fungi were sensitive to all five EOs, especially to cinnamon and thyme, compared with the control,^23^ and their result is widely supported by other researchers.^45–47^

On the other hand, Purkait et al.^48^ reported that the synergistic effect of clove and cinnamon oils exhibited cytotoxicity against *Allium cepa* roots, with an IC_50_ value exceeding 2000 ppm. Their study aimed to assess the oils’ synergistic antimicrobial and antioxidant activities, comparing the average root length of the control group with a 100% growth, to the decrease in root growth of treated groups at various concentrations.^48^ Our results showed that the IC_50_ value of clove EO was determined to be 324.7 ppm (Figure 6). Additionally, our SIM-based LC–MS analysis suggests that clove EO at concentration of approximately 325 ppm is required to trigger a 50% reduction in AFB1 in treated peanuts. The difference between these two IC_50_ values is attributed to the cytotoxicity of clove EO, which may have more metabolic targets against fungal cells than floral cells.

Furthermore, our high *R* ^2^ of 0.9914 for the inhibition curve (Figure 6) demonstrates a strong correlation between the concentration of clove EO and the recorded AFB1 abundance of contaminated seeds. Similarly, in our previous study,^6^ we observed a high *R* ^2^ of 0.9957 in the relationship between the clove EO concentration used to treat *A. flavus* mycelium grown on Petri plates, and the concentration of AFB1 detected using the ammonia vapor (AV) and coconut milk agar (CMA) methods. The only difference between our two studies is that we quantified AFB1 in artificially inoculated peanut seeds in our current study, whereas seeds were naturally infected in the previous study. While a 10% decrease in AFB1 concentration was observed on coconut milk agar plates treated with 500 ppm of clove EO in our earlier study,^6^ in this study, we found that ∼70% of AFB1 inhibition occurred in 500 ppm of clove EO in artificially inoculated seeds. Compared to our previous study, the difference in the decrease of AFB1 (%) activity at 500 ppm of clove EO observed in this study is likely due to the intricate nature of the peanut seed matrix rather than the mycelium and spores. According to previous reports, clove EO inhibits AFB1 production due to its abundance of bioactive components, including eugenol, eugenyl acetate, and β-caryophyllene.^49,50^ Kumar Pandey et al.^49^ and Rana et al.^50^ have both confirmed that these compounds possess antifungal and antimicrobial properties, which we believe could effectively inhibit the growth and metabolic activities of aflatoxin-producing fungi, including *Aspergillus* spp. Santos et al.^51^ found eugenol, eugenol acetate, and β-caryophyllene in clove EO by hydrodistillation, gas chromatography (GC), and differential scanning calorimetry (DSC). Achar et al.^6^ used gas chromatography–mass spectrometry (GC–MS) analysis to identify that eugenol (83.25%) is the primary bioactive ingredient in clove EO. Additionally, Pinto et al.^52^ obtained similar results using GC–MS and found a high eugenol content (85.3%). Phenols, such as thymol, carvacrol, and eugenol, possess a system of delocalized electrons and may also reduce the pH gradient across the cytoplasmic membrane by acting as proton exchangers. The collapse of the proton motive force and depletion of the ATP pool, resulting from this effect, can lead to the leakage of iron and intracellular constituents, eventually causing cell death. The mitochondria in *A. flavus* also play critical roles in aflatoxin biosynthesis, and plant-based natural components may disrupt them, thereby ceasing the formation of acetyl-CoA that feeds the aflatoxin biosynthesis pathway, ultimately leading to the inhibition of aflatoxin biosynthesis.^53^

## 5 Conclusions

In summary, we found that as the concentration of clove EO increases, there is a significant decrease in mycelial growth and sporulation in artificially inoculated organic peanut seeds. Relative to the control, the higher concentrations of clove EO in treated seeds resulted in lower production of AFB1, with diminishing effectiveness, such that less than 1% of AFB1 was detected at 2000 ppm of clove EO-treated seeds compared to the control (0 ppm). No previous reports have indicated that clove EO has been tested as an antifungal and antiaflatoxigenic agent against *A. flavus*, more specifically in organic peanuts in Georgia. Furthermore, we demonstrated for the first time the potential of clove EO to reduce AFB1 production by *A. flavus* in organic peanuts. These findings highlight clove EO as a promising, eco-friendly biological control agent and a potential alternative to synthetic chemicals for managing aflatoxin contamination in peanut production. Moreover, the use of clove EO could contribute to reducing AFB1 levels in peanuts and peanut-based products in our food chain, thereby enhancing food safety. Nevertheless, further research is required to evaluate the practicality and cost-effectiveness of clove EO applications for organic farming on a large scale. Our current field trials, in progress, may provide additional data on its efficacy as a biological control agent within management strategies against *A. flavus* in organic peanut production.
